# Optimal Coil Orientation for Transcranial Magnetic Stimulation

**DOI:** 10.1371/journal.pone.0060358

**Published:** 2013-04-11

**Authors:** Lars Richter, Gunnar Neumann, Stephen Oung, Achim Schweikard, Peter Trillenberg

**Affiliations:** 1 Institute for Robotics and Cognitive Systems, University of Lübeck, Lübeck, Germany; 2 Graduate School for Computing in Medicine and Life Sciences, University of Lübeck, Lübeck, Germany; 3 Department of Neurology, University Hospital of Schleswig-Holstein, Lübeck, Germany; Katholieke Universiteit Leuven, Belgium

## Abstract

We study the impact of coil orientation on the motor threshold (MT) and present an optimal coil orientation for stimulation of the foot. The result can be compared to results of models that predict this orientation from electrodynamic properties of the media in the skull and from orientations of cells, respectively. We used a robotized TMS system for precise coil placement and recorded motor-evoked potentials with surface electrodes on the abductor hallucis muscle of the right foot in 8 healthy control subjects. First, we performed a hot-spot search in standard (lateral) orientation and then rotated the coil in steps of 10° or 20°. At each step we estimated the MT. For navigated stimulation and for correlation with the underlying anatomy a structural MRI scan was obtained. Optimal coil orientation was 33.1±18.3° anteriorly in relation to the standard lateral orientation. In this orientation the threshold was 54±18% in units of maximum stimulator output. There was a significant difference of 8.0±5.9% between the MTs at optimal and at standard orientation. The optimal coil orientations were significantly correlated with the direction perpendicular to the postcentral gyrus (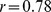). Robotized TMS facilitates sufficiently precise coil positioning and orientation to study even small variations of the MT with coil orientation. The deviations from standard orientation are more closely matched by models based on field propagation in media than by models based on orientations of pyramidal cells.

## Introduction

Transcranial magnetic stimulation (TMS) non-invasively activates cortical neurons that in turn, when targeting the motor cortex, cause muscle contraction [1], [2]. The strength of the contraction can be recorded as a motor evoked potential (MEP) by using surface electrodes over this muscle.

The neurons are stimulated by a current distribution that is induced by a transient magnetic field. This magnetic field is generated by a short current pulse that is sent through a stimulation coil. For a given position of the stimulation coil on the head, the magnetic field penetrates the whole skull and induces a current density distribution that is characterized by a direction and magnitude that both vary within the skull. These quantities are determined by the coil position and geometry, and by the geometry and electrical conductivity of the tissue. An MEP will ensue if the current density at the position of a target neuron, that directly or indirectly is wired with the muscle, exceeds a threshold value to depolarize the axon membrane [3], [4].

For a figure-8 coil, the largest current density is attained directly below the center of the coil. Thus, it is assumed that the center of the coil indicates the position of the target cells that control a given muscle. However, this is only valid when the conductivity inhomogeneities are ignored and the threshold is minimal with respect to surrounding coil positions.

**Figure 1:**
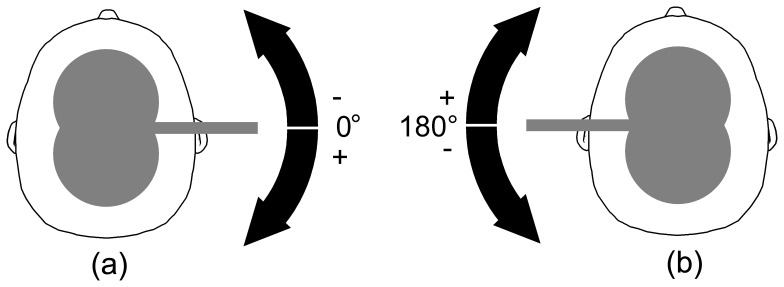
Definition of coil orientation angles for stimulation of the right foot. (a) As standard reference for stimulation of the right foot a lateral right-to-left coil orientation was used. We indicated this coil orientation with 0° and used it in session 1 as reference; (b) The opposite coil orientation (indicated with 180°) to the standard coil orientation. The arrows show the rotational direction.

In addition to coil position, coil orientation also influences thresholds and MEP amplitudes in TMS. In clinical routine and experimental treatments with repetitive TMS, this is considered by recommending standard orientations [5]. Such standard orientations are, for instance, posterior-lateral for the hand muscles [6]–[8] and perpendicular to the interhemispheric cleft ( =  lateral) for leg muscles [9] (cf. Figure 1a). In (brain) research, the coil orientation is commonly adjusted to the coil orientation (and position) yielding in the highest MEP amplitude. However, this results in additional stimulation/session time and is therefore commonly neglected in clinical applications [5].

The recordings of the motor threshold (MT) as a function of coil orientation that are available in the literature are not very precise with steps at least as large as 45°. Muscles in the hand have been the most frequent targets for motor cortex research [6], [10]. In addition to the MT, the MEP amplitude [11] or latency were investigated [12], [13]. Furthermore, brain mapping with different current directions was studied [14], [15]. However, optimal directions were then calculated by interpolation or inferred from fits of sinusoids to the results. For the stimulation of the hand the coil current was at an angle of 45° with respect to the sagittal direction for optimal stimulation [6], [11].

For stimulation of the leg motor area (anterior tibial muscle), Terao et al. additionally investigated the MEP amplitudes and latencies for different coil orientations with 45°-steps. They confirmed that a lateral coil orientation produced the highest MEP amplitudes and shortest latencies [16].

Implications of optimal coil orientation for the underlying physiology were discussed by Fox et al. who proposed a cortical column cosine (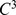) model to describe the interaction of the induced electric field direction and the cerebral cortex [17]. They calculate the effective electric field based on the cortical orientation in relation to the absolute electric field. Furthermore, it has been hypothesized that neurons are stimulated only if their axons curve away from the current induced in the tissue [17].

In addition to this rather simplified model, recent realistic simulations take the topography of the human cortex into consideration [18], [19]. These simulations show that for identical coil currents, the magnitude of the induced current in the brain critically depends on the orientation of the coil relative to gyri and sulci [18].

The cortical representation of the abductor hallucis muscle (AHM) is one of the deepest structures in M1. Thus, it is located directly at the medial wall of the hemisphere, making the estimation of the alignment of the cortical columns easier than for the hand motor cortex which has the shape of a knob and thus, columns in many different directions [20]. In contrast to the AHM, the cortical representation of the anterior tibial muscle (ATM), which has been targeted in previous studies on the motor leg area [16], is located more closely to the surface of the brain. Thus, estimation of its alignment is less straight-forward.

For TMS in general, precise coil positioning is a prerequisite for accurate recordings. Neuro-navigation combines a three-dimensional (3D) scan of the patient’s head with a real time tracking system and is applied with increasing frequency [21]. After registration and tracking of coil and head, the TMS coil can be positioned based on the 3D head scan. This allows for precise coil positioning and target localization [22], [23]. Robotized TMS, as a recent development, combines the benefits of neuro-navigation with automation [24], therefore allowing for precise coil positioning and reproducibility. Active motion compensation keeps the coil at the selected target during stimulation, without the need for head fixation or motion restriction [25].

In this study, we investigate the impact of coil orientation with respect to the alignment of the underlying precentral gyrus on the stimulation result. In contrast and in addition to previous studies,

- we use a robotized TMS system to precisely place and rotate the TMS coil and to ensure that the coil maintains tangential orientation to the scalp;
- we use small coil-rotation steps of 10° to accurately measure the optimal coil orientation angle;
- we record MEPs on the AHM as the alignment of the precentral gyrus for the AHM is easier to estimate in contrast to the ATM or even the hand area; and
- we use structural MRI images to locate the stimulation target in the precentral gyrus and to estimate its alignment.

Applying this setup, we are able to show that.

1. the optimal coil orientation angle for stimulation of the foot is 30° posterior than the common standard coil orientation;
2. the inter-subject variability is much smaller than reported in previous studies; and
3. the optimal coil orientation angle correlates to the alignment of the precentral gyrus which will be discussed with respect to model and simulations.

Preliminary results of this study have been already presented as a conference abstract [26].

## Methods

### Experimental Setup

We used an MC-B70 Butterfly coil with a slight bend and a MagPro X100 stimulator with MagOption (MagVenture A/S, Farum, Denmark) for focused biphasic stimulation. To reach sufficiently high stimulation intensity the ‘power mode’ of the device was used, which allowed a 1.4 times higher stimulation power compared to the standard mode. Recording of MEPs was accomplished using a 2-channel DanTec Keypoint Portable (Alpine Biomed Aps, Skovlunde, Denmark) with surface electrodes. For placing and holding the coil precisely, we used a robotized system that is based on an Adept Viper s850 serial six joint robot (Adept Technology, Inc., Livermoore, CA, USA) and a Polaris infrared stereo-optical tracking system (Northern Digital Inc., Waterloo, Ontario, Canada) [24], [27]. For navigated stimulation, a T1-weighted structural magnetic resonance imaging (MRI) scan was obtained prior to recording (Achieva 3T, Philips Medical, Amsterdam, The Netherlands). The following scanning settings were applied: TR/TE = 8.1/3.7, with a volume of 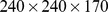 voxel and a voxel size of 1 mm in each spatial axis. This MRI-scan was also used for analysis of the orientation of the gyrus that was stimulated. The shape of head was extracted from the MRI-scan. During stimulation a set of markers, attached to the head, was tracked for a change in marker position and orientation by the camera system as aforementioned. The correlation between marker position/orientation and the MRI-scan was established by retracing the head surface with a pointer prior to the stimulations. As a double-stage correlation algorithm is used based on three landmarks and more than 400 surface points, the average co-registration error remains below 0.4 mm [24]. The result of the tracking of head movements was fed into the control of the robot, which maintained a constant coil position during one series of stimulations. For different series it was guaranteed that only the coil orientation was varied. The overall coil positioning accuracy of robotized TMS with this setup is 1.8 mm on average [28].

This study was approved by the local ethics committee:

Ethikkommission der Universität zu Lübeck (Ethics committee of the University of Luebeck)

Ratzeburger Allee 160, Building 21, D-23562 Luebeck

Chairman: Prof. Dr. med. Dr. phil. H. Raspe

The recordings were performed on eight healthy male subjects with no history of neurologic disease aged 24 to 31 years after written informed consent had been obtained.

### Motor Threshold Estimation

As the MEP amplitude has a high variance [29], we employed the MT as a more robust quantitative measure for cortical excitability in relation to the coil orientation.

In general, the MT is defined as the stimulation intensity at which a muscle contraction occurs with a probability of 50%. In our case, we classified the MEP signal as a muscle contraction if the base-to-negative peak amplitude exceeded 50 *μV* (for the resting muscle). The MT is reported as a percentage of maximum stimulator output (MSO).

To accurately and systematically estimate the MT for a given coil orientation, we employed the threshold hunting method [30]. This adaptive method calculates the most likely intensity being the MT based on the success/failure at previously used intensities. To this end, it applies a maximum likelihood estimation, based on best PEST (parameter estimation by sequential testing) [31], to calculate the most likely MT [30], [32]. For the next stimulation pulse this intensity is used for measuring the MEP. The step size is reduced in each iteration to gradually approach the MT. It was reported that on average 17 stimulation pulses are required to calculate a reliable MT with this method [33]. For even more conservative results, we took 30 pulses as termination criteria for each MT calculation. We employed the TMS Motor Threshold Assessment Tool, a freeware program to perform this threshold hunting [34].

### Transcranial Magnetic Stimulation

We stimulated the motor cortex of the left hemisphere and recorded MEPs on the AHM of the right foot.

For each subject, two stimulation sessions were performed on different days. The sessions were designed such that coil orientation in session 1 is opposite to session 2. As we were using biphasic stimulation, we expected two threshold minima (at slightly different stimulation intensities) occurring at coil orientations differing by 180°. This way, we could further verify the optimal coil orientation in terms of stability within the subjects. For session 1 we used a right-to-left coil orientation as reference (Figure 1a), which is the current standard orientation, and for session 2 we used a left-to-right coil orientation as reference (Figure 1b).

For each session, we first performed a hot-spot search. We used the median in MEP amplitude of 5 subsequent stimulations, in standard orientation and opposite orientation, respectively. A grid of positions with a distance of 1 cm was used and stimuli were applied with fixed stimulation intensity (usually 70% of maximal stimulator output). The hot-spot was defined as the optimal stimulation site that was surrounded by four other stimulation points with smaller MEP amplitudes. Thus, depending on the starting point, at least five stimulation points were used. Consequently, not less than 25 MEPs were employed for each hot-spot definition.

Subsequently, we placed the coil at the hot-spot again and rotated the coil to different orientations. At each coil orientation, we performed a motor threshold estimation and afterwards rotated the coil to the next orientation. For session 1, we rotated the coil clockwise from −20° to 80°, where 0° denotes the reference (left-to-right) coil orientation. For session 2, we used orientations from 160° to 280°, where 180° denotes the right-to-left coil orientation, used as reference for session 2. In the range of the minimum MT, we used orientations in steps of 10°. In the outer range, we employed a step size of 20° so that a clear curve with a distinct minimum could be recorded.

Note that we split the investigation into two sessions to limit effects of varying vigilance. For the same reason, we used steps of 20° and 10° to not unnecessarily prolong the session, although we are able to use very small coil rotation steps with the robotized TMS system. With our setup, each session lasted approximately 1.5 hours. In sessions with duration less than 1.5 h, we repeated the motor threshold estimation for the first orientation at the end of the stimulation series. The difference between the two recordings serves as a measure for coil positioning accuracy and for the influence of changes in vigilance.

The coil orientations and the sessions were randomized. The MT estimation was performed in a double-blind way: The robot operator but not subject and investigator were aware of the current coil orientation.

### Analysis

#### Stability of recordings and MT curves

First of all, we analyzed the stability of the MT recordings based on the repeated MT estimations at the end of the session. Stable MTs in the first and in the repeated estimation suggest that the measurements have been performed correctly and accurately. In addition to the average MT difference, we calculated the correlation coefficient *r* between first and repeated MT estimations. Subsequently, we evaluated the recorded MT curves for each subject.

#### Optimal coil orientation and statistical analysis

Based on the recorded MTs, we estimated the optimal coil orientation angle. Minimal thresholds and thresholds at standard orientation were compared with a repeated measures t-test, using MATLAB (The MathWorks, Inc., Natick, MA, USA). Similarly, the optimal coil orientation angle was compared to the standard coil orientation angle.

Since motor thresholds vary considerably between subjects (for example due to skalp cortex distance), we also calculate the standardized MT values for each subject. In our case, we compute the standardized MT value for subject *s* and coil orientation *α* as:
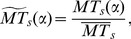
(1)where 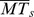 represents the average MT for subject *s*.

To quantify effect sizes in our results, we calculated *Cohens d* [35] for the comparison of the minimum thresholds vs. thresholds in standard orientation. Given that a within subjects comparison is characterized, the use of the groups standard deviation results in a conservative estimate for *d*.

#### Curve fitting

In general, the waveform of biphasic stimulation is cosine-shaped with the second halfwave having the largest amplitude. Thus, the second phase in biphasic stimulation is physiologically most effective [5]. However, also the initial rising phase contributes to the stimulation effect. As the slope in this phase is smaller in amplitude than in the second phase [36], its impact is less effective. Consequently, we can expect that a coil orientation opposite to the optimal coil orientation will also have a stimulation effect. However, the effect will be smaller in relation to the optimal coil orientation. From the previous studies investigating the effect of coil orientation, even though they were very coarse, we know that also at coil orientations 90° from the suggested best orientation, stimulation effects can be recorded [6], [10], [16]. In relation to the best orientation and to its opposite, the effects at 90°, however, have been smaller.

In total, we can therefore expect a sinusoidal for biphasic stimulation with two minima between coil orientation angle 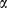 and motor threshold. This sinusoid should have period 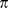 (as opposed to 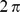 which would be trivial) with the global minimum roughly at 0 (standard orientation) and the second minimum approximately at 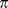 (left-to-right orientation). Due to different slopes of the coil current pulse, the MT at 0 should be smaller than the MT at 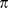. Consequently, a second sinusoid with period 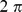 should be added to express the orientation dependent amplitude change.

Therefore, the sinusoidal relation should have the form
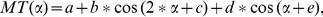
(2)where 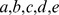 are constant factors.

For every subject and for the group averages, we fitted the experimental data to this sinusoidal relation with nonlinear regression and estimated the error of the fit. The fitting was performed using MATLAB.

As a quantitative measure for the goodness of the sinusoidal fitting, we used the coefficient of determination 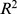. It is defined as:
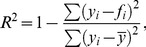
(3)where 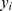 represents the estimated MTs for a given coil rotation *i*, 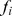 is the value of the sinusoidal fit at 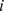, and 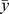 symbolizes the mean of the estimated MTs.

#### Correlation to gyrus orientation

**Figure 2:**
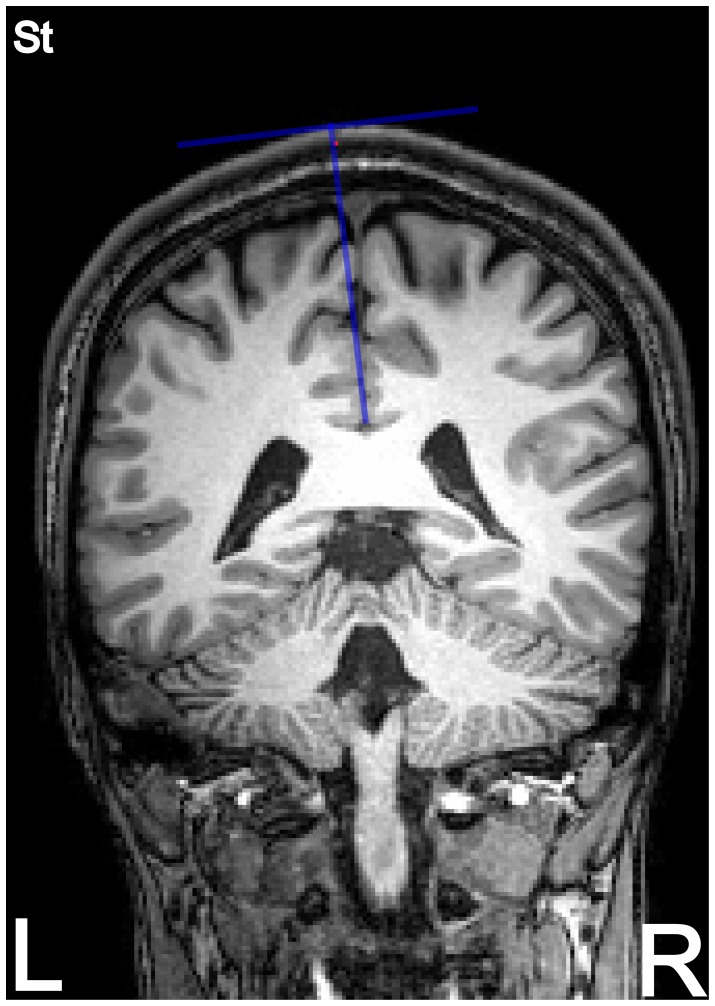
Coronal view of a structural MRI at hot-spot level. From the hot-spot (red dot) at the skull the normal to the skalp surface is extended into the brain. The intersection of this line with the precentral gyrus is used for later evaluation of the angle of the precentral gyrus with respect to the interhemispheric cleft (cf. Figure 8).

Finally, we investigated whether the orientation of the underlying gyrus that has been stimulated correlates with the optimal coil orientation. To this end, we used a transversal plane of the individual MRI images at that level where the foot area is suggested in the precentral gyrus. With our TMS navigation and robot control software, we are able to precisely determine the coil’s spatial position and orientation with respect to the subject’s head contour and thus with the subject’s brain anatomy (based on the structural MRIs). For determination of the stimulation point in the precentral gyrus, we first selected a coronal view in the navigation software. We then went through the coronal slices from the back to the front until we met the plane where the hot-spot on the skull was located. Then, we followed the normal of the head surface at the hot spot into the brain until we reached the gyrus at the medial surface of the brain (cf. Figure 2). At this level, we estimated the angle of the precentral gyrus with the interhemispheric cleft in a transverse image. This estimation was done by visual inspection. If the anterior and the posterior bank of the precentral gyrus form different angles with the interhemispheric cleft, the mean of the two angles was chosen. Even though the central sulcus did not reach the interhemispheric fissure in 3 out of our seven subjects, we were able to estimate a bisection line. We extrapolated the bisection line in these cases until it intersects with the interhemispheric cleft. The angles were estimated in a blinded fashion by two examiners and the average gyrus angle estimate of each subject was used. For further analysis, we compared this angle to the optimal coil orientation. We calculated the correlation between gyrus orientation and optimal coil orientation angle and estimated the significance of correlation coefficients *r* with a repeated measures t-test on 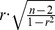, where *n* denotes the number of subjects.

## Results

The stimulator’s ‘power mode’ was mostly well accepted by the subjects. However, two subjects felt inconvenient due to the strong muscle twitching and the impact on the skin. Therefore, subjects ‘Ti’ and ‘Pa’ only participated in one of the two sessions. For subject ‘La’ we performed session 1 twice (‘La1’ and ‘La2’). As for both trials no minimum could be found, subject ‘La’ was excluded from further analysis. Thus, we have six full recordings for each session for evaluation.

### Stability of Recordings and MT Curves

In total, we were able to repeat the MT estimation of the first coil orientation at the end of the stimulation in 8 sessions. On average, the MT difference was 1.88±0.83% of MSO. The correlation coefficient *r* between first and repeated MT estimation was 0.99. This very small MT difference and the very high correlation coefficient support that the effect of fatigue was minimal and that we positioned the coil very accurately.

The hot-spot was located close to midline over the medial lip of the precentral gyrus for all subjects and for both sessions which also suggest accurate coil positioning.

**Figure 3:**
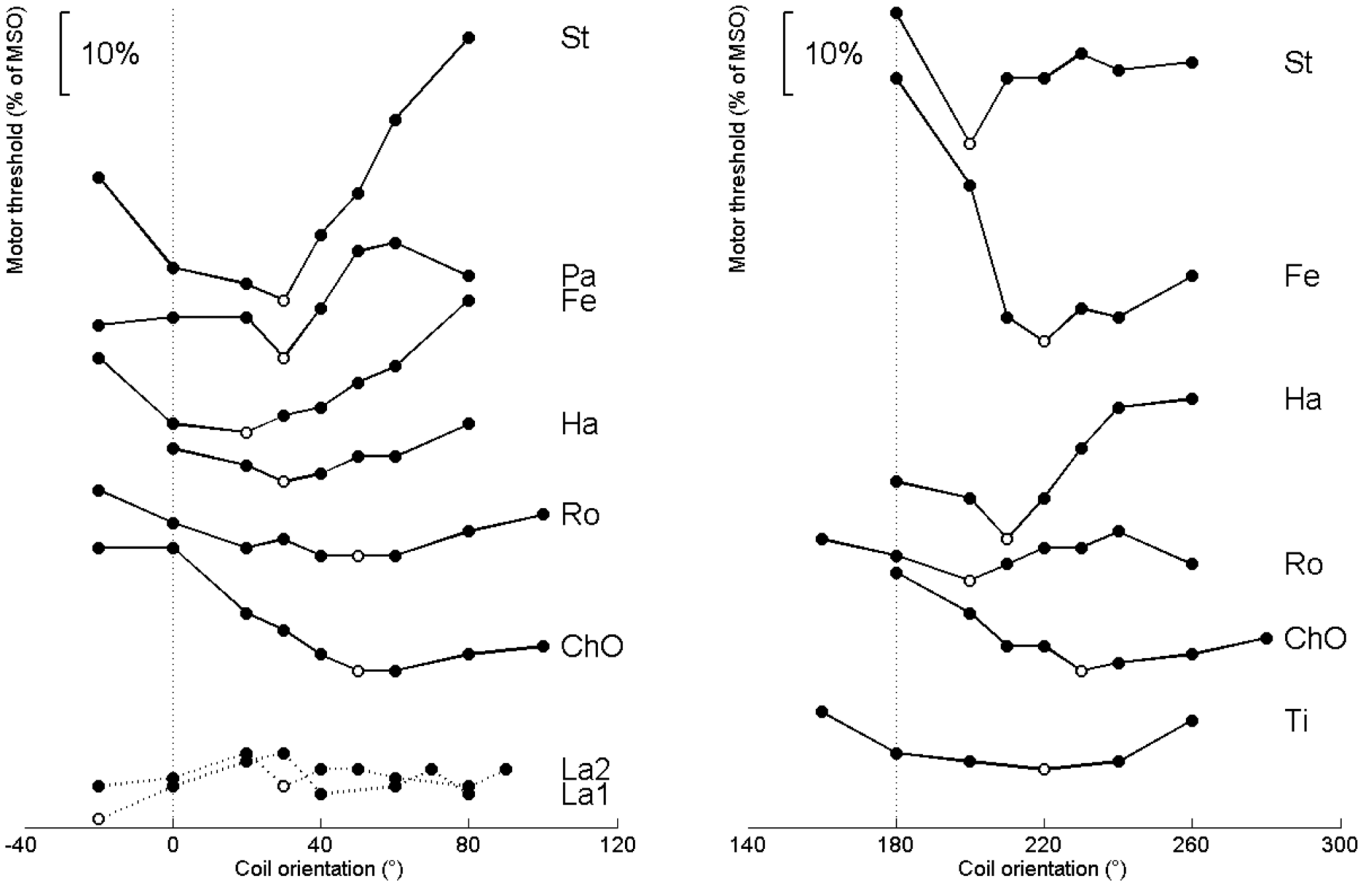
Recordings of threshold vs. coil orientation for each subject (labeled with acronyms). The reference coil orientation for each session is indicated with a dotted vertical line. The minimum values for each subject are highlighted with open circles. In the left panel the recordings for session 1 with the standard coil orientation as reference are shown. The right plot shows the recordings for session 2. Note that the individual curves are vertically shifted for best presentability. Subject ‘La’ was excluded from further analysis as no clear minimum could be estimated in two sessions. For the sake of completeness subject ‘La’ is still shown in this figure.

Figure 3 illustrates the motor thresholds with respect to the coil orientation for sessions 1 and 2 for all subjects. Note that all the curves show a distinct minimum: First, the recordings decrease monotonically toward the minimum and then increase monotonically to the more sagittal orientations of the coil. In all sessions and all subjects the MT minimum was between 20° and 50° clockwise from the reference coil orientations at 0° and 180°, respectively.

### Optimal Coil Orientation and Statistical Analysis

**Figure 4:**
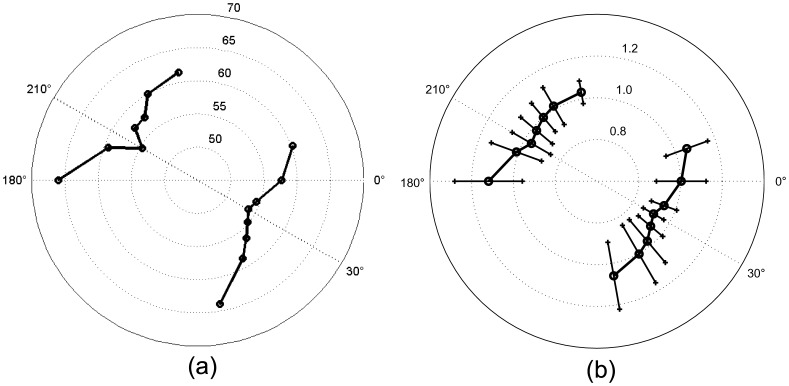
Polar plots of the motor thresholds averaged throughout subjects. (a) shows the average of the real MTs (cf. Table 1); (b) shows the average of the standardized values with error bars (cf. Table 2). For both subplots: Recordings on the right side represent the findings for the standard coil orientation. Recordings in the left part were obtained with the coil handle towards the left hemisphere. Threshold minima occur in opposite positions as expected for a biphasic pulse.

Figure 4a shows the motor thresholds averaged throughout subjects as a polar plot. In this figure the two opposite minima are clearly demonstrated. Both minima are rotated 30° clockwise from the reference orientation. The minimum for session 1 is located at 30° and results in a MT of 53.8%±17.7% of MSO. In contrast, the standard orientation results in an averaged MT of 57.6%±16.0% of MSO. The same trend applies to the standardized MT values. Figure 4b displays the averaged standardized MTs with error bars.

For the opposite coil orientation (coil handle towards the left hemisphere), the minimum is located at 210° and results in a motor threshold of 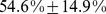 of MSO. In contrast, the average MT at 180° (reference orientation) was 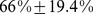 of MSO. The optimal coil orientation angle showed a significant difference to the standard coil orientation angle (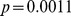 and 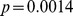 for sessions 1 and 2, respectively).

Table 1 summarizes the motor thresholds averaged throughout subjects with its standard deviations for all coil orientations for sessions 1 and 2. Table 2 shows the averaged values for the standardized MTs.

**Table 1:** Motor thresholds averaged throughout subjects.

	Session 1								Session 2						
	−20°	0°	20°	30°	40°	50°	60°	80°	180°	200°	210°	220°	230°	240°	260°
Mean	60.25	57.6	54.4	**53.8**	54.8	56.4	58.6	64	66	59.3	**54.6**	57.3	57.4	60	61.5
SD	21.1	16.0	17.9	**17.7**	21.3	23.9	26.7	29.8	19.4	15.6	**14.9**	13.6	17.2	15.4	17.0
RMS	63.0	59.4	56.7	**56.1**	58.0	60.3	63.3	69.3	68.3	61.0	**56.2**	58.7	59.4	61.6	63.4
Min	42	38	35	**33**	30	28	28	30	45	40	**36**	36	33	34	35
Max	84	73	71	**69**	77	82	91	101	94	81	**73**	73	76	74	75

Motor thresholds averaged throughout subjects with standard deviations (SD) in relation to the coil orientation for both sessions. Furthermore, the root mean square (RMS) values, and the minimum (Min) and maximum (Max) thresholds are presented for each coil orientation. The MTs are presented in % of MSO.

**Table 2:** Standardized motor thresholds averaged throughout subjects.

	Session 1								Session 2						
	−20°	0°	20°	30°	40°	50°	60°	80°	180°	200°	210°	220°	230°	240°	260°
Mean	1.06	1.00	0.94	**0.91**	0.93	0.97	1.00	1.06	1.12	1.01	**0.96**	0.98	1.00	1.02	1.03
SD	0.11	0.12	0.06	**0.06**	0.08	0.13	0.16	0.16	0.16	0.13	**0.11**	0.10	0.09	0.10	0.05
RMS	1.06	1.01	0.94	**0.91**	0.94	0.98	1.01	1.07	1.13	1.02	**0.97**	0.98	1.00	1.02	1.03
Min	0.93	0.91	0.86	**0.84**	0.83	0.79	0.79	0.85	0.93	0.84	**0.82**	0.88	0.94	0.93	0.97
Max	1.22	1.22	1.03	**1.00**	1.02	1.13	1.17	1.30	1.34	1.15	**1.10**	1.15	1.15	1.20	1.10

Standardized motor thresholds averaged throughout subjects with standard deviations (SD) in relation to the coil orientation for both sessions. Furthermore, the root mean square (RMS) values, and the minimum (Min) and maximum (Max) thresholds are presented for each coil orientation. The MTs are presented as standardized values.

The average coil orientation for the minimum threshold was 33.1°±18.3° for session 1 and 213.3°±12.1° for session 2. The mean MT difference between optimal orientation and reference orientation was 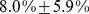 of MSO and 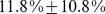 of MSO for sessions 1 and 2, respectively. This difference in motor threshold amplitude was significant for both sessions (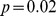 and 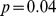 for sessions 1 and 2, respectively).

With our results, *Cohens d* was 0.47. As this is commonly regarded as an intermediate effect size, it indicates that our results are relevant even though the number of subjects is rather small.

### Curve Fitting

After fitting the average recordings to the model sinusoidal, the resulting curve had the form:
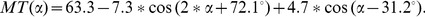
(4)


The coefficient of determination, 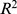, of the sinusoidal fitting was 0.86 which is significant at the 0.01 level (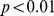). The average 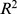 for each subject was 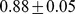. On average, the sinusoidal minimum was located at 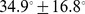 and 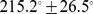 for session 1 and 2, respectively.

**Figure 5:**
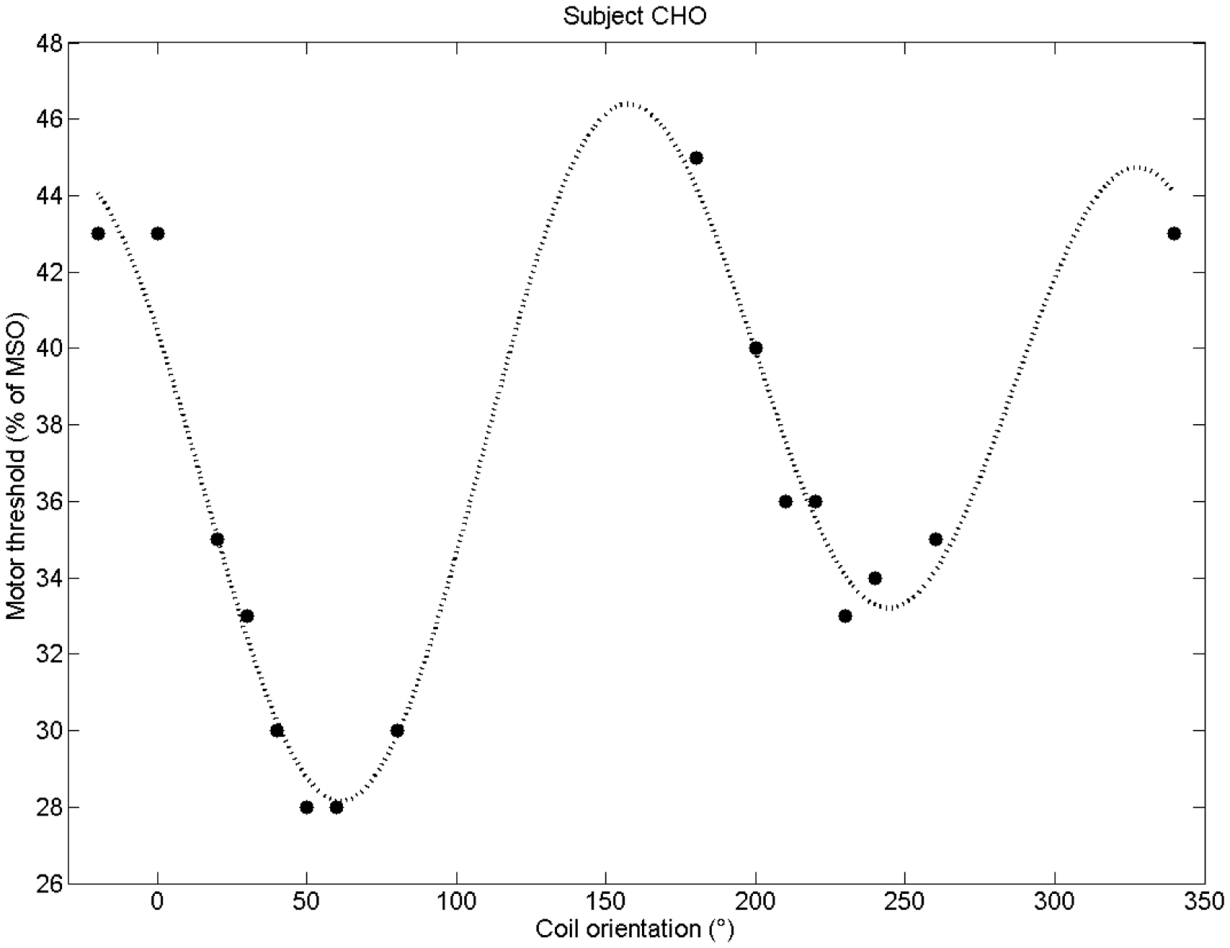
The motor threshold recordings for both sessions of subject ‘ChO’. A sinusoidal curve (dotted line) was fitted to the recordings. The global minimum at the optimal coil orientation is slightly smaller than the local minimum at the opposite coil orientation. The standard coil orientation (at 0°), however, is clearly not optimal.

Figure 5 shows the computed sinusoidal curve for subject ‘ChO’ as an example. The sinusoid smoothly fits the recordings as expected. The local minimum opposite to the optimal orientation is slightly larger than the global minimum.

### Correlation to Gyrus Orientation

**Figure 6:**
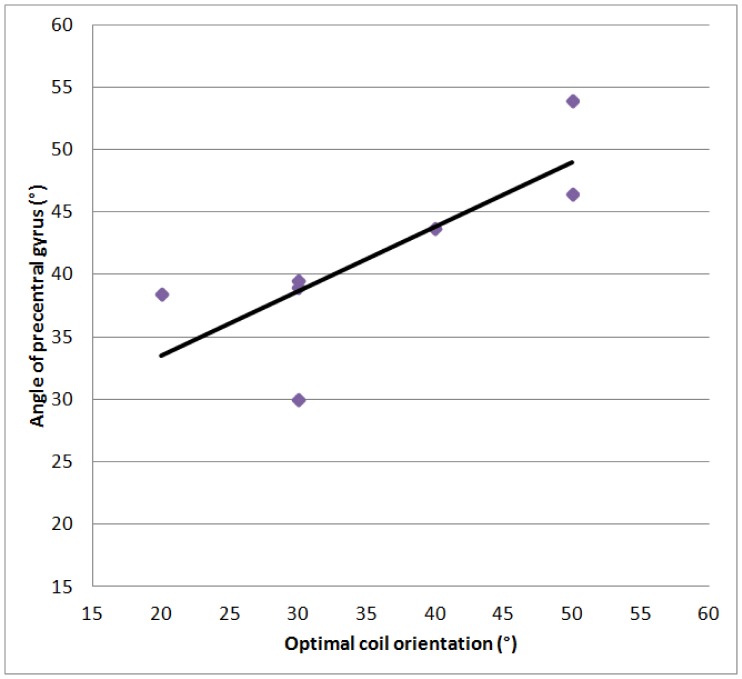
Correlation between optimal coil orienation and angle of the precentral gyrus. The individual values are also listed in Table 3.

The orientation of the gyrus underneath the hot-spot estimated in the MRI scans is presented in Table 3. Additionally, the optimal coil orientation angles are reported for the subjects. The correlation coefficient (Pearson’s *r*) between angle of the precentral gyrus and the optimal coil orientation was 0.78. The correlation was therefore significant (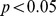). This correlation is also plotted in Figure 6.

**Table 3:** Optimal coil orientation and angle of the precentral gyrus.

Subject	opt. coil rot.	angle of gyrus
ChO	40°	46.5°
Fe	20°	38.5°
Ha	30°	39.5°
Pa	30°	30°
Ro	50°	54°
St	30°	39°
Ti	40°	43.75°

Optimal coil orientation and angle of the precentral gyrus with respect to the interhemispheric cleft at cortical hot-spot position for each subject.

## Discussion

The monotonicity in our measurements documents that we have identified reliable minima with our setup. However, for one subject, we have not been able to estimate a clear minimum in two sessions. A reason for this might be the variation in structural anatomy, especially in the primary motor cortex [37]. Although there is some variation of optimal coil angle between the subjects, the lateral coil orientation is clearly not optimal. The optimal coil rotation for stimulation of the right foot (abductor hallucis muscle) deviates approximately 30° from the standard coil orientation. The MT difference of optimal coil rotation to standard rotation was 8.0% and 11.8% of MSO, respectively.

Furthermore, our recordings support the assumption of a sinusoidal relationship between coil orientation and stimulation outcome – in this case with the motor threshold as quantitative parameter. The fit result (cf. Figure 5) however mainly relies on the recordings around the minima. Due to our setup, only a few recordings for the maxima region exist. With more recordings in the maxima regions the sinusoidal curve may slightly change. However, the general trend – due to the minima – should remain. Note that this model also fits well to the data presented by Balslev et al. for the hand region [10].

Although the optimal coil orientation in our recordings differed between subjects, the inter-individual variability was essentially smaller than reported by Balslev et al. for the hand region. In our study the variability was 30° whereas Balslev et al. reported a variability of 63° [10]. In contrast to their study, we used precise coil orientations in small steps. Balslev et al. used the principle component analysis (PCA) to compute optimal coil orientations for each subject based on recordings with coarse rotation steps of 45° [10]. Even though most of their results are convincing, their PCA results for their subject 11 in session 2 are not compelling as the optimal orientation (thus, a threshold minimum) calculated by PCA was 88.5° whereas a local maximum was at 90°. Thus the large scatter of optimal coil angle in this study could be an artifact due to the technique that was used to obtain more precise results from a coarse grid of angles studied.

With respect to the interaction of electrical field and cells, Fox et al. suggest that the effect of the induced electrical field depends on the orientation of the cortical column [17]. The electric field must be aligned with the column to be effective. If the electric field is perpendicular to the column, the electric field is completely ineffective [17]. In its original presentation the model focuses on axons of pyramidal cells. These axons are oriented in parallel to the axis of cortical columns, thus perpendicularly to the surface of the brain. In this case, a piece of cortex that is oriented parallel to the surface of the head could not be stimulated at all. A parasagittal piece of cortex could be stimulated optimally with left to right orientation of a butterfly coil and not at all with an anterior-posterior orientation of the coil. However, the stimulation of pyramidal axons is thought to give rise to D-waves (“direct”) in contrast to I-waves (“indirect”) that arise from the stimulation of interneurons. In surface recordings, these types of excitation can be identified from their latency. For stimulation of the anterior tibial muscle with a double cone coil it was shown that exclusively I-waves are elicited [16]. With a butterfly coil in optimal orientation there was a preference for I-waves over D-waves suggesting an important contribution of interneurons. The orientation of the dendrites and axons of interneurons is less clear than for pyramidal cells. If there was no preference in their orientation there would be no variation of threshold with coil orientation. If the interneurons were oriented perpendicularly to the axis of the cortical column a parasagittal piece of cortex would be optimally stimulated with the coil handle oriented in anterior-posterior direction and not at all with a left-to-right orientation. At least data from sensory cortex of the rat suggest that some interneurons have axons and dendrites oriented along the cortical column and some others have an isotropic shape [38]. This would imply that optimal coil orientation for I-wave and for D-wave stimulation would coincide but that there is no absolutely ineffective coil orientation.

**Figure 7:**
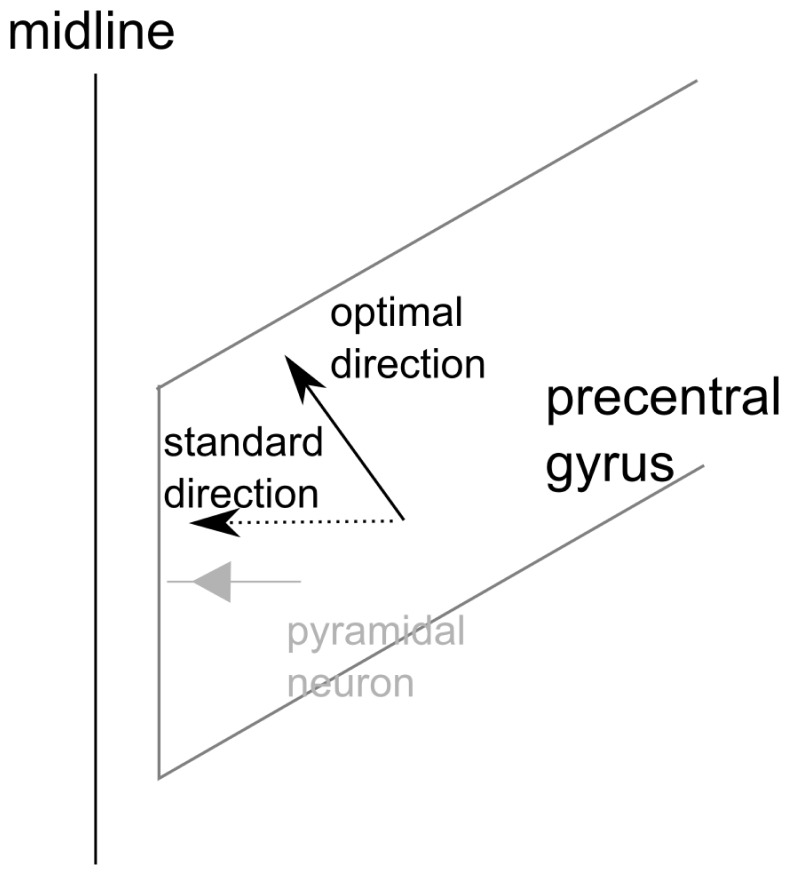
Schematic view on the precentral gyrus at the medial wall. The standard current direction is in lateral direction which is in alignment with the orientation of pyramidal neurons at the medial wall. The optimal current direction in our results is approximately 30° rotated. This would be in alignment with pyramidal neurons at the posterior (or anterior) wall.

In addition to the uncertainty of the orientation of the cells influenced by TMS the orientation there is also some degree of uncertainty about the orientation of the cortical columns. We assumed that the target area for the stimulation of the leg is located at the medial surface of the brain (see Figure 7). This is suggested by focal intraoperative electrical stimulation [39]. Activations elicited in functional imaging studies are usually presented in views from medial aspects of the brain [40], but coronal views also show some activation on the paramedian cranial surface of the brain [41]. If there is also some spread anteriorly the columns would be oriented in anterior-posterior direction. There might even be a twofold representation of the foot as this has been shown for the hand [37]. The foot was chosen for our study, because we assumed that the orientation of the relevant cortex would at least be more unambiguous than for the hand. Since the hand motor cortex forms a knob the columns form a bundle of directions that spans a significant part of a sphere [42].

**Figure 8:**
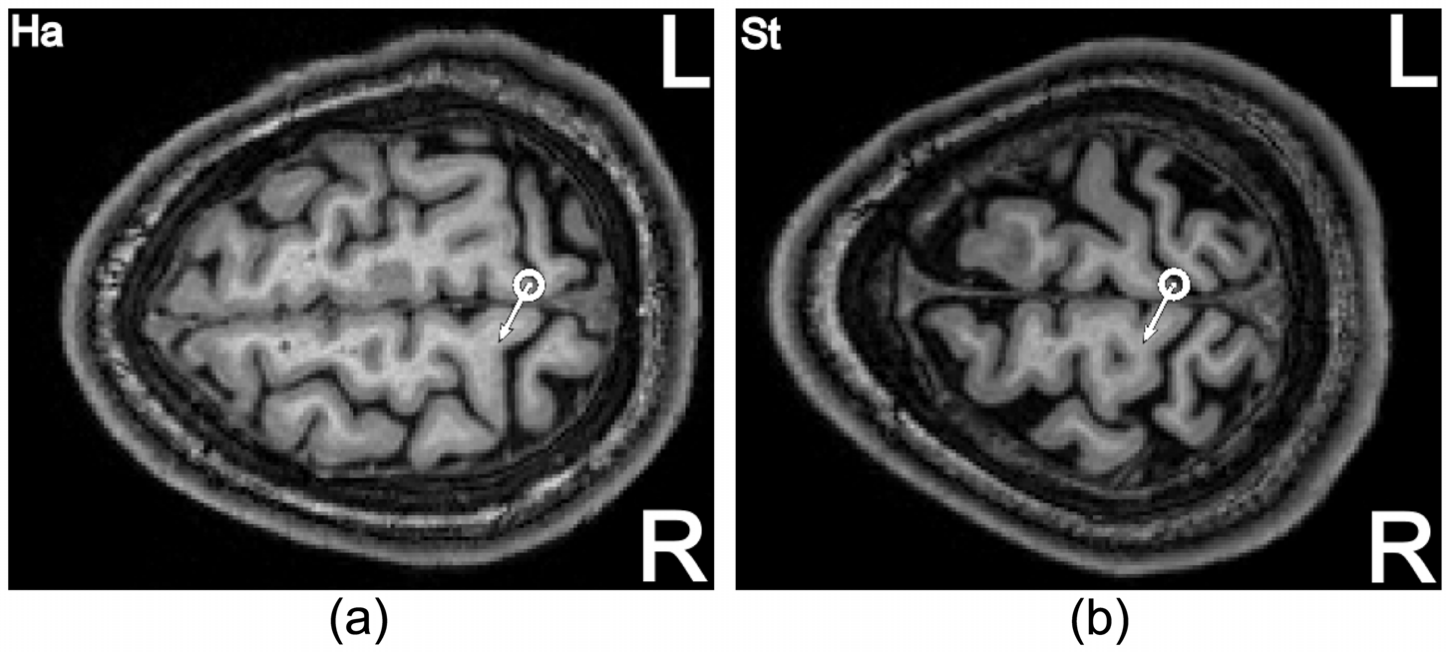
Hot-spot (dot with surrounding circle) for two subjects projected in the MRI images in a transversal view. An area of the precentral gyrus at the edge to the central sulcus and close to the interhemispheric cleft is in focus for stimulation. The white arrows denote the found optimal coil orientation angle for the individual subject.

Models that simulate the propagation of electromagnetic fields in the complex distribution of different conductivities in the skull predict optimal stimulation for a coil handle perpendicular to the crown of the gyrus [18]. Note that in this model the interaction of the stimulus with a particular neuron is not addressed. Nonetheless, this prediction is recovered in our experimental data. In Figure 8, we exemplarily show the hot-spot for two subjects projected in the MRI images in a transversal view. When projecting the hot-spot from the scalp surface down to the cortex, we see that an area of the precentral gyrus at the edge to the central sulcus and close to the interhemispheric cleft is the focus for stimulation. The optimal coil orientations in our recordings are shown as white arrows. They are almost perpendicular to the precentral gyrus at the hot-spot as also indicated by the significant correlation (cf. Table 3). This is consistent with the simulations by Thielscher et al. [18].

In conclusion, we can assume that the precentral gyrus orientation is the key factor for an optimal current direction and therefore for an optimal coil orientation. This is in analogy with the work by Terao et al., where the shortest MEP latencies were found when stimulating almost perpendicular to the underlying gyrus [43]. However, they targeted the hand area of the motor cortex. Therefore, inducing an electric field perpendicular to the precentral gyrus might be the reason why the optimal coil orientation for the foot is almost the same as the standard orientation for the hand region [11].

As a more direct test of the macroscopic models optimal directions for hand and foot stimulation could be determined in the same subjects. If these were identical an interaction with a larger structure can be assumed that in turn influences smaller parts of cortex. The robotized TMS system is a powerful and sufficient tool for this purpose as it can rotate the coil very precisely and in small steps while keeping the coil in a tangential orientation to the head.
